# Supplementary material for: Revealing the immune perturbation of black phosphorus nanomaterials to macrophages by understanding the protein corona
Source: Nat Commun. 2018 Jun 26;9:2480. doi: 10.1038/s41467-018-04873-7 (PMC6018659; doi:10.1038/s41467-018-04873-7)
Supplement: Supplementary file 1 — Supplementary Information [file 41467_2018_4873_MOESM1_ESM.pdf]

Supplementary Information

Revealing the immune perturbation of black phosphorus nanomaterials to macrophages by understanding the protein corona

Jianbin Mo[1, 2, #], Qingyun Xie[3, #], Wei Wei[1, 2, ★] and Jing Zhao[1, ★]

[1] State Key Laboratory of Coordination Chemistry, Institute of Chemistry and BioMedical Sciences, School of Chemistry and Chemical Engineering, Nanjing University, Nanjing, 210093, P. R. China

[2] State Key Laboratory of Pharmaceutical Biotechnology, School of Life Sciences, Nanjing University, Nanjing 210093, China.

[3] Department of Orthopedics, Chengdu Military General Hospital, Chengdu, 610083, P. R. China.

# J.M. and Q.X contributed equally to the work.

★ E-mail: weiwei@nju.edu.cn, jingzhao@nju.edu.cn

Results

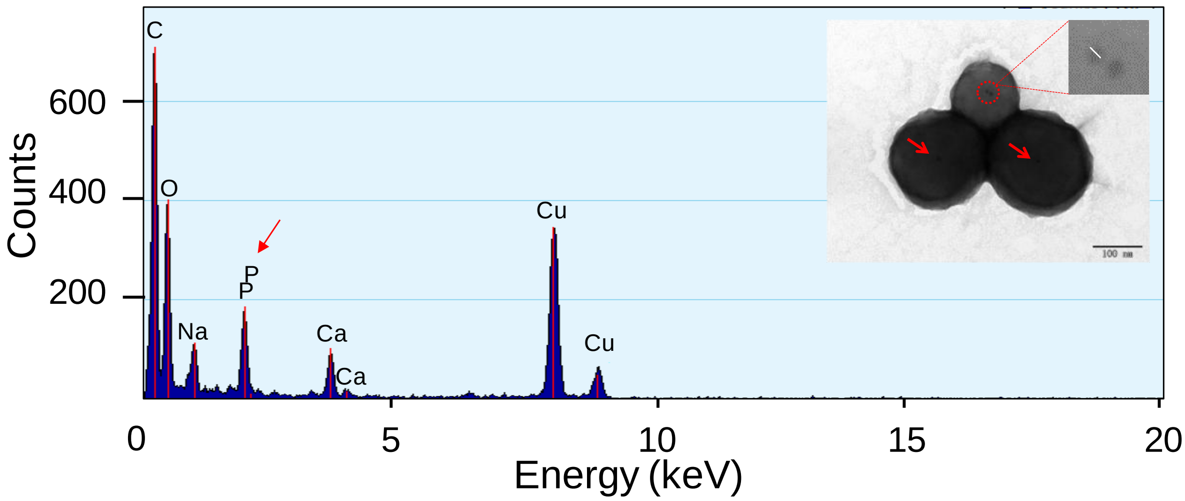

Supplementary Figure 1. Energy dispersive X-ray spectroscopic analysis of BPQD-corona complexes. Inset: magnified TEM image of BPQD-corona complexes. Scale bar: 5.32 nm

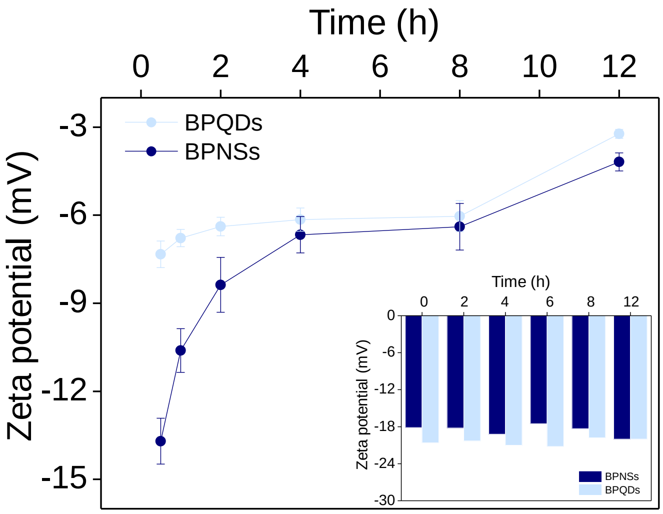

Supplementary Figure 2. Zeta-potential of BP nanomaterials and BP-corona complexes. Values expressed were means ±SDs of triplicates.

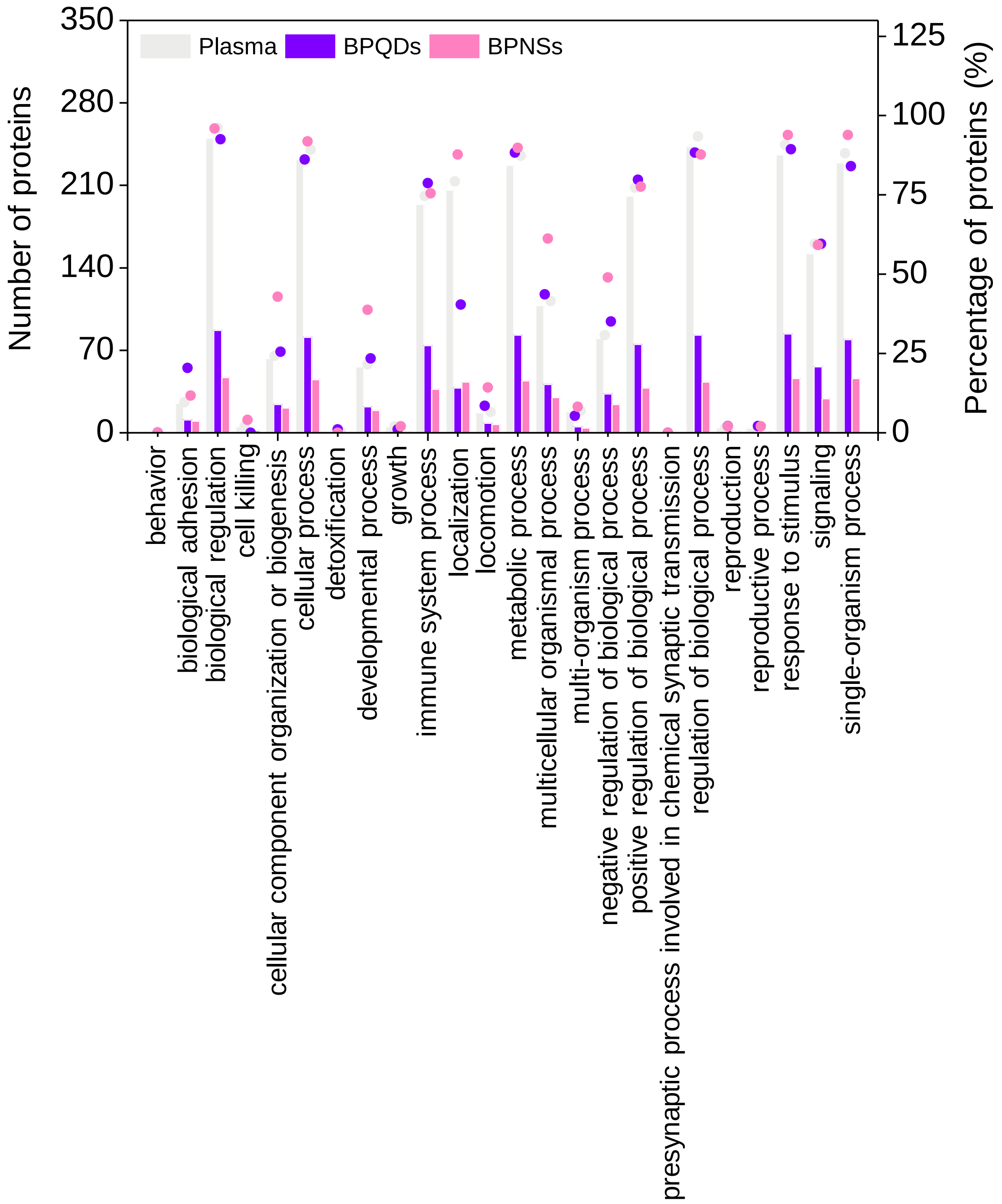

Supplementary Figure 3. Bioinformatic classification of identified plasma protein according to biological process by gene ontology analysis. The histogram represents the number of proteins. The scatter diagram represents the percentage of protein.

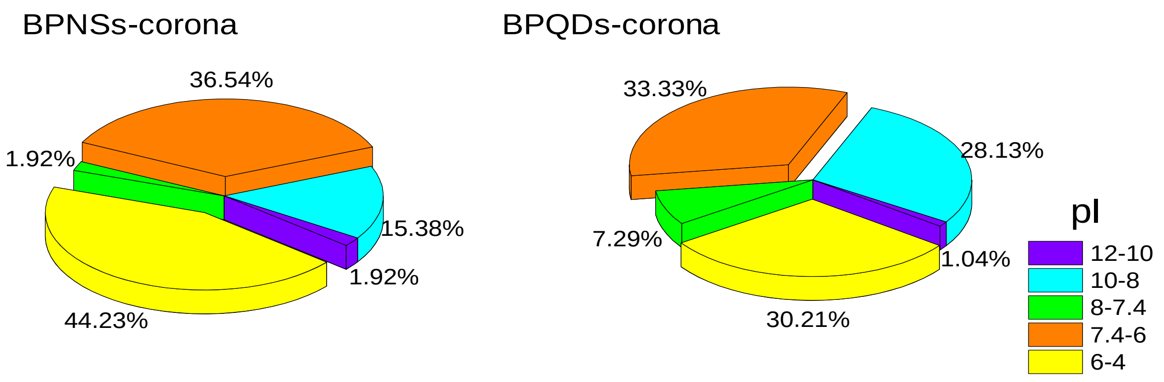

Supplementary Figure 4. Proteins were classified according to their isolelectric point (pI), as analyzed by LC-MS/MS. The bulged part highlighted the proteins with pI < 7.4.

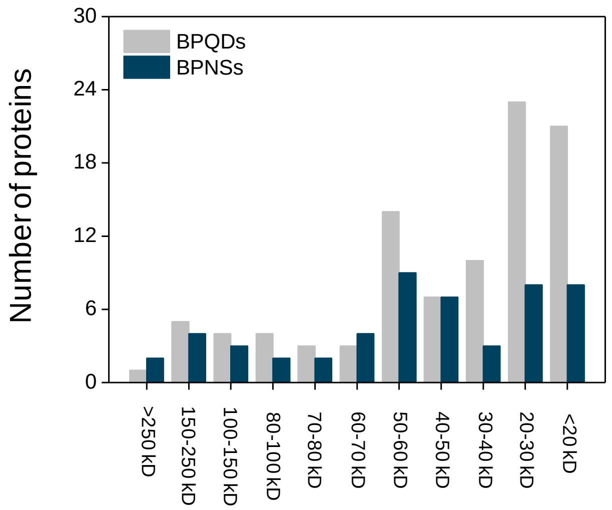

Supplementary Figure 5. Proteins were classified according to their Molecular Weight (MW), as analyzed by LC-MS/MS.

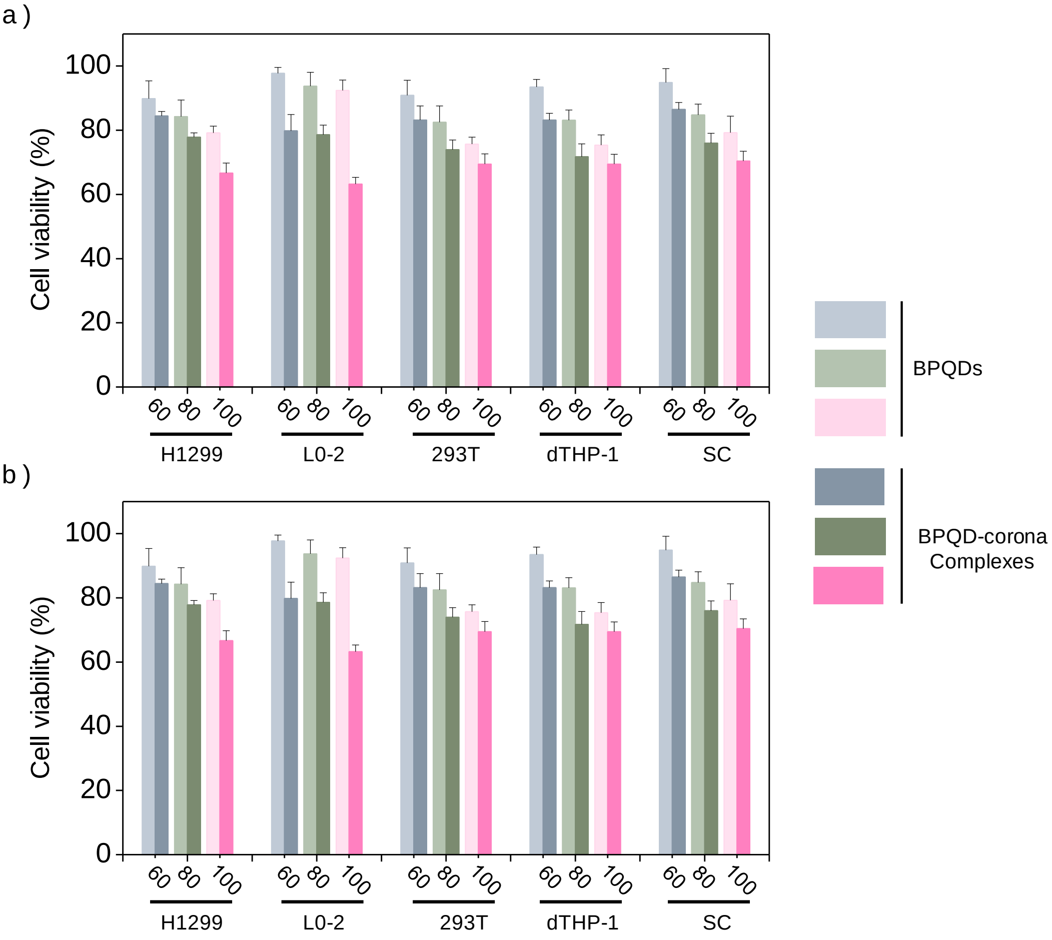

Supplementary Figure 6. Cytotoxicity of BPQD- and BPQD-corona complexes against different kinds of cells. (a) Cells were treated with different concentrations of BPQD- and BPQD-corona complexes (60, 80 and 100 μg ml^{-1}) in serum-free media for 48 h. (b) Cells were treated with different concentrations of BPQD- and BPQD-corona complexes (60, 80 and 100 μg ml^{-1}) in serum media for 48 h. Values expressed were means ± SDs of triplicates.

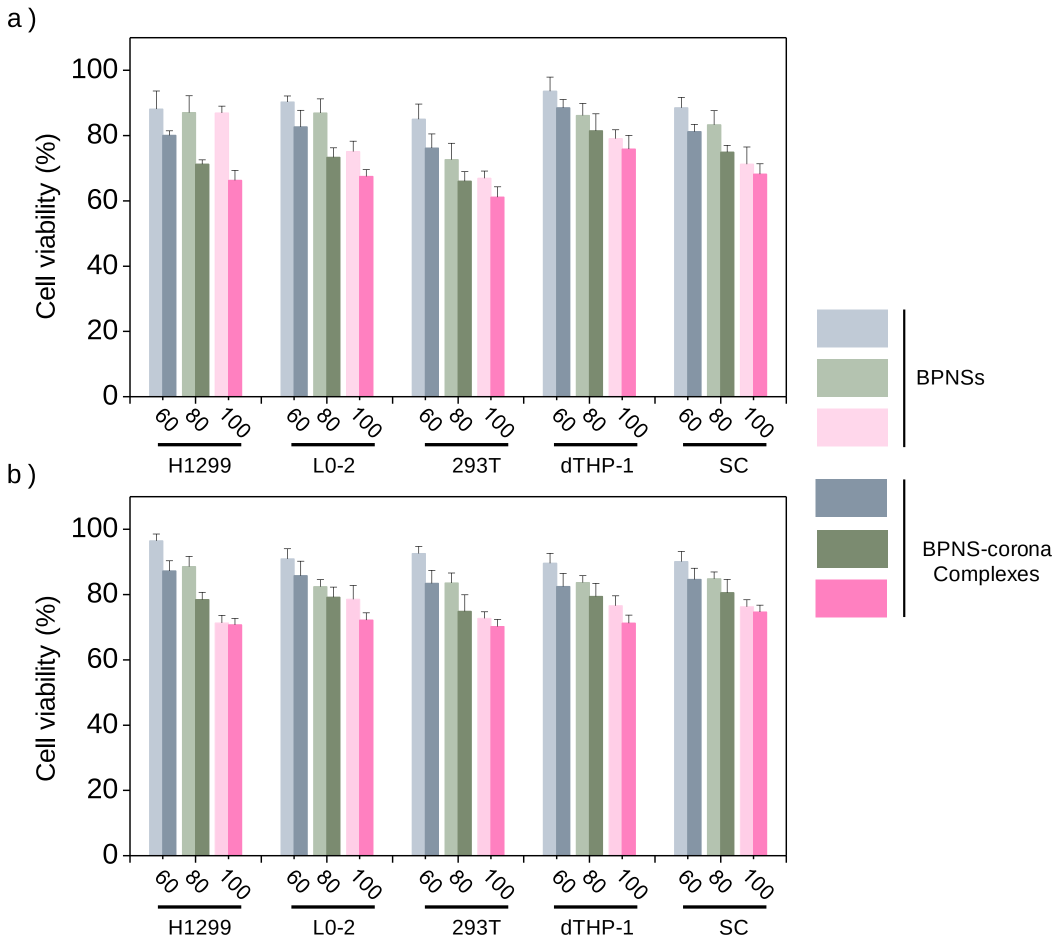

Supplementary Figure 7. Cytotoxicity of BPNS- and BPNS-corona complexes against different kinds of cells. (a) Cells were treated with different concentrations of BPNS- and BPNS-corona complexes (60, 80 and 100 μg ml^{-1}) in serum-free media for 48 h. (b) Cells were treated with different concentrations of BPNS- and BPNS-corona complexes (60, 80 and 100 μg ml^{-1}) in serum media for 48 h. Values expressed were means ± SDs of triplicates.

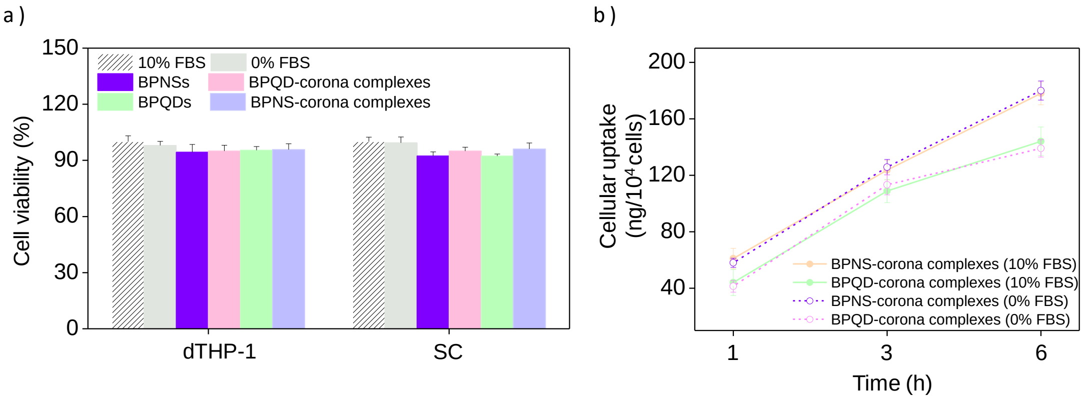

Supplementary Figure 8. Effect of serum-free medium on macrophage cells. (a) Cytotoxicity of BP nanomaterials and corona complexes. Cells were treated with 100 μg ml^{-1} nanomaterials for 6 h in serum-free media. Medium with 0% FBS and 10% FBS were set as control. Values expressed were means ± SDs of triplicates. (b) Comparison of the cellular uptake of BP-corona complexes in different medium. Macrophage-like dTHP-1 cells were treated with 150 μg ml^{-1} corona complexes for 1, 3 and 6 h. Values expressed were means ± SDs of triplicates.

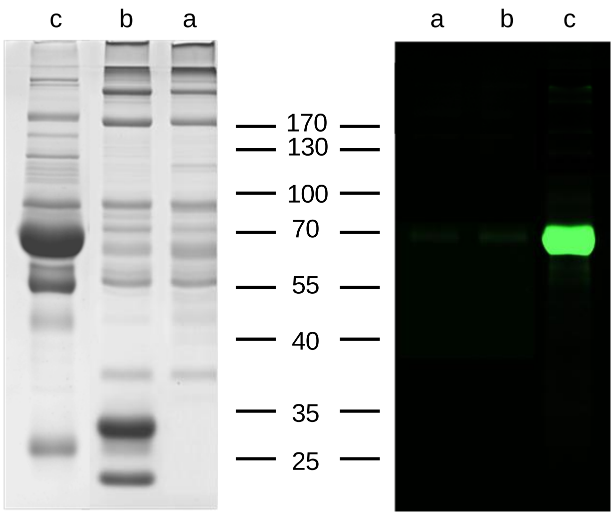

Supplementary Figure 9. SDS-PAGE gel of protein obtained from (a) BPQD-corona complexes and (b) BPNS-corona complexes incubated with mixture protein fluid (plasma protein and 10 μg HAS-FITC protein). Lane c was set as the control. The molecular weights of the proteins in the standard ladder are reported on the middle.

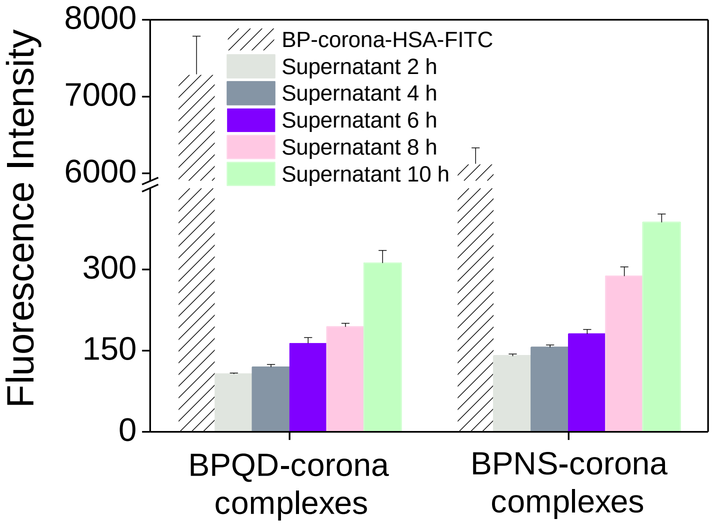

Supplementary Figure 10. Stability of fluorescent BP-corona complexes. After remove the BP-corona-HSA-FITC complexes, the fluorescence of supernatant were detected at different time. The fluorescence of BP-corona-HAS-FITC suspensions was also measured as control. Values expressed were means ± SDs of triplicates.

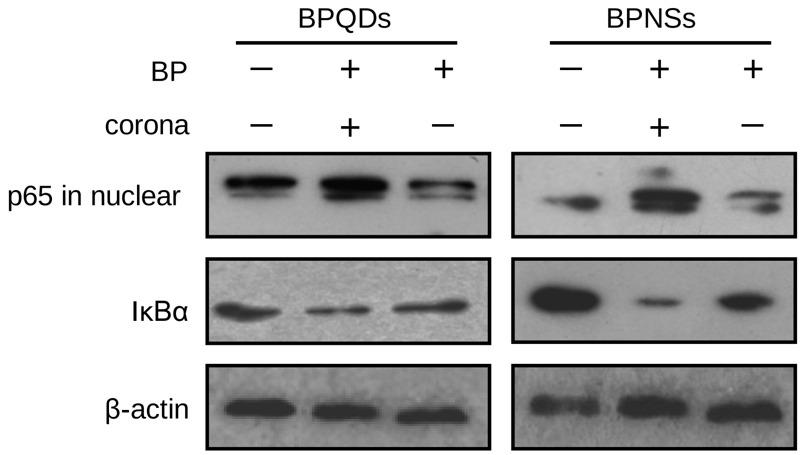

Supplementary Figure 11. Activation of NF-κB pathways by BP nanomaterials and corona complexes. Macrophage-like dTHP-1 cells were treated with nanomaterials at 100 μg ml^{-1} for 6h.

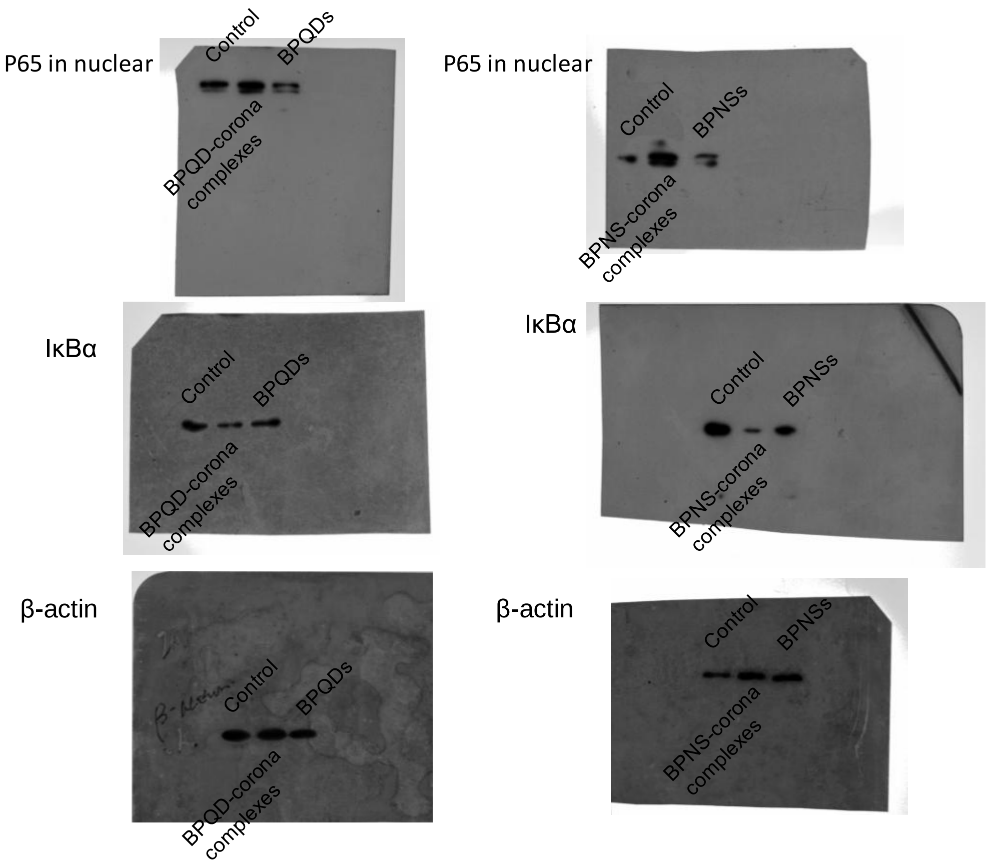

Supplementary Figure 12. Uncropped images of Western blots from Supplementary Figure 11.

Supplementary Table 1 DLS analysis of BP nanomaterials and BP-corona complexes

	**PBS**		**Serum-free Media**	
Nanomaterial	Particle size (nm)	PDI	Particle size (nm)	PDI
BPNSs	338.4±2.3	0.28	341.2±3.3	0.24
BPNS-corona complex	365.3±5.9	0.19	359±2.8	0.28
BPQDs	5.6±1.4	0.22	5.8±1.1	0.25
BPQD-corona complex	362.5±5.6	0.20	371.2±4.7	0.31

Supplementary Table 2. List of detailed immune system processes of plasma protein identified by LC-MS/MS after exposure to BPQDs.

Uniprot accession number	Protein ID	Unique PepCount	Cover Percent	Biologial process	ref
P02768	Serum albumin	52	75.53%	Toxic substance binding; Rresponse to external stimulus; Response to stress	1, b
B4E1B2	Serotransferrin	34	46.31%	Immune response; Response to stress	b
P01024	Complement C3	27	17.44%	Complement activation; Immune response; Neutrophil degranulation; Inflammatory response	2,3
P01023	Alpha-2-macroglobulin	26	21.30%	Negative Regulation of complement activation; Lectin pathway	4
P00738	Haptoglobin	24	41.13%	Regulation of intestinal barrier function	5
P02671	Fibrinogen alpha chain	19	23.21%	Facilitate the immune response via both innate and T-cell mediated pathways	6
P01871	Ig mu chain C region	15	32.08%	Adaptive immune response; Complement activation, classical pathway; Positive regulation of B cell activation	7
P08603	Complement factor H	14	13.89%	Complement activation	8
P02647	Apolipoprotein A-I	13	43.45%	Negative regulation of Inflammatory response; Negative regulation of response to cytokine stimulus	9
V9HW68	Epididymis luminal protein 214	12	26.17%	Immunoglobulin mediated immune response; Fc-gamma receptor signaling pathway; Fc-epsilon receptor signaling pathway	b
Q6N089	Uncharacterized protein	11	24.58%	Fc-gamma receptor signaling pathway;	b

S6B291	IgG H chain	11	24.89%	Immunoglobulin mediated immune response; Fc-gamma receptor signaling pathway; Fc-epsilon receptor signaling pathway; Immunoglobulin mediated immune response	b
Q96K68	SNC73 protein (SNC73)	11	25.30%	Immune effector process; Activation of immune response; Complement activation	b
Q6ZVX0	Protein Tro alpha1 H,myeloma	10	23.61%	Positive Regulation of immune system process; Immune effector process; Cellular response to stimulus; Immune response-regulating signaling pathway	b
Q8NCL6	Ig alpha-1 chain C region	10	25.76%	Positive Regulation of immune system process; Complement activation	b
V9HVY1	Epididymis secretory sperm binding protein Li	10	19.14%	Humoral immune response; Response to cytokine	b
Q8N355	IGL@ protein	9	51.71%	Positive Regulation of immune system process; Immune response-regulating cell surface receptor signaling pathway involved in phagocytosis; Humoral immune response	b
Q6MZQ6	Putative uncharacterized protein	9	22.95%	Positive Regulation of immune system process; Immune response-regulating cell surface receptor signaling pathway involved in phagocytosis; Complement activation	b
A0A0G2JPR0	Complement C4-A	9	5.91%	Complement activation; Inflammatory response	a
Q03591	Complement factor H-related protein 1	9	27.88%	Complement activation; Regulation of complement activation	10
P02790	Hemopexin	9	21.21%	Positive regulation of immunoglobulin production; Positive regulation of humoral immune response mediated by circulating immunoglobulin	a
P02679	Fibrinogen gamma chain	9	18.32%	Regulation of response to stimulus	b
Q8N5F4	IGL@ protein	8	44.64%	Positive Regulation of immune system process; Immune response-regulating cell surface receptor signaling pathway involved in phagocytosis; Humoral immune response	b
Q6GMV8	Uncharacterized protein	8	47.01%	Immune response-regulating cell surface receptor signaling pathway involved in phagocytosis; Complement activation; Immune response-regulating signaling pathway; Adaptive immune response; Iymphocyte mediated immunity	b
Q6P5S8	IGK@ protein	8	45.76%	Humoral immune response; Complement activation; Positive Regulation of immune response	b
V9HW34	Epididymis luminal protein 213	8	48.94%	Positive Regulation of immune response	b
Q5EBM2	Uncharacterized protein	8	16.18%	Immune response-regulating cell surface receptor signaling pathway involved in phagocytosis; Complement activation; Adaptive immune response	b

Q6MZU6	Putative uncharacterized protein DKFZp686C15213	8	15.52%	Humoral immune response; Immune response-regulating signaling pathway; Leukocyte mediated immunity	b
B4E1D8	C4b-binding protein alpha chain	8	14.37%	Complement activation; Negative regulation of immune effector process; Negative regulation of Adaptive immune response	b
Q9NPP6	Immunoglobulin heavy chain variant	8	25.96%	Humoral immune response; Positive Regulation of immune response	b
A2KBC6	Anti-FactorVIII scFv	8	31.93%	Immune response-regulating cell surface receptor signaling pathway involved in phagocytosis; Defense response; Complement activation	b
A0A5E4	Uncharacterized protein	7	40.00%	Humoral immune response; Complement activation; Immune response-regulating signaling pathway	b
Q5FWF9	IGL@ protein	7	38.36%	Positive Regulation of immune system process; Immune response-regulating cell surface receptor signaling pathway involved in phagocytosis; Humoral immune response	b
Q7Z2U7	Uncharacterized protein	7	40.17%	Adaptive immune response; Humoral immune response; Complement activation; Immune response-regulating signaling pathway	b
P01859	Ig gamma-2 chain C region	7	24.23%	B cell receptor signaling pathway; Complement activation; Defense response to bacterium; Positive regulation of B cell activation	a
Q6GMW4	IGL@ protein	7	41.20%	Positive Regulation of immune system process; Immune response-regulating cell surface receptor signaling pathway involved in phagocytosis; Humoral immune response	b
Q6NS95	IGL@ protein	7	44.02%	Positive Regulation of immune system process; Immune response-regulating cell surface receptor signaling pathway involved in phagocytosis; Humoral immune response	b
Q6MZX7	Putative uncharacterized protein DKFZp686M24218	7	17.23%	Leukocyte mediated immunity; Adaptive immune response; Complement activation; Immune response-regulating signaling pathway	b
S6BAR0	IgG L chain	6	36.57%	Immune response-regulating cell surface receptor signaling pathway involved in phagocytosis; Complement activation; Leukocyte mediated immunity	b
O43866	CD5 antigen-like	6	17.58%	Cellular defense response; Immune system process; Inflammatory response	11
S6BAP0	IgG H chain	6	24.02%	Humoral immune response; Complement activation; Immune response-regulating signaling pathway	b
C0JYY2	Apolipoprotein B (Including Ag(X) antigen)	6	2.65%	Regulation of nitric oxide biosynthetic process	b
Q65ZC9	Single-chain Fv	6	16.67%	Immune response-regulating cell surface receptor	b

				signaling pathway involved in phagocytosis; Complement activation; Immune response-regulating signaling pathway	
P04196	Histidine-rich glycoprotein	6	12.95%	Immunoglobulin binding	12
S6BGE0	IgG H chain	5	21.33%	Humoral immune response; Complement activation; Immune response-regulating signaling pathway	b
S6BAM6	IgG H chain	5	19.75%	Humoral immune response; Complement activation; Immune response-regulating signaling pathway	b
A0A0B4J1X5	Immunoglobulin heavy variable 3-74	5	30.77%	Innate immune response	a
V9HWF6	Alpha-1-acid glycoprotein	5	19.90%	Negative regulation of cytokine production	b
S6BGF5	IgG H chain	4	21.67%	Humoral immune response; Complement activation; Immune response-regulating signaling pathway;	b
A0N5G1	Rheumatoid factor C6 light chain	4	25.86%	Immune response-regulating cell surface receptor signaling pathway involved in phagocytosis	b
P01591	Immunoglobulin J chain	4	30.19%	Immune response; Innate immune response	13,14
P19652	Alpha-1-acid glycoprotein 2	4	21.39%	Regulation of immune system process	a
P36980	Complement factor H-related protein 2	4	15.19%	Regulation of complement activation	a
S6AWF4	IgG L chain	3	23.89%	Immune response-regulating cell surface receptor signaling pathway involved in phagocytosis; Complement activation; Leukocyte mediated immunity	b
P02749	Beta-2-glycoprotein 1	3	12.17%	Regulation of fibrinolysis	15
V9GYM3	Apolipoprotein A-II	3	39.85%	Positive regulation of cytokine production; Cytokine production involved in immune response	b
A0A068LRX1	Ig heavy chain variable region	3	18.00%	Humoral immune response; Complement activation; Immune response-regulating signaling pathway	b
A0A0J9YX35	Uncharacterized protein	3	15.38%	Leukocyte mediated immunity; Adaptive immune response; Complement activation; Immune response-regulating signaling pathway	b
A0N7I9	F5-20	3	14.63%	Humoral immune response; Complement activation; Immune response-regulating signaling pathway	b
P02746	Complement C1q subcomponent subunit B	3	9.49%	Complement activation; Innate immune response	a
P02747	Complement C1q subcomponent subunit C	3	17.55%	Complement activation; Immune response; Innate immune response	16
P02760	Protein AMBP	3	11.93%	Regulation of immune response	b
Q13784	APOA4 protein	3	11.07%	Response to oxidative stress; Innate immune response; Organ or tissue specific immune response	b
A0A0X9TD47	MS-D1 light chain variable region	2	14.95%	Humoral immune response; Complement activation; Immune response-regulating signaling pathway	b
A0N5G5	Rheumatoid factor D5 light chain	2	14.41%	Humoral immune response; Complement activation; Immune response-regulating signaling pathway	b
A0A075B7B8	Protein IGHV3OR16-12	2	18.80%	B cell receptor signaling pathway; Innate immune response; Complement activation	a
A0A087X232	Complement C1s subcomponent	2	4.84%	Complement activation	a

A0A0X9UWL5	GCT-A5 light chain variable region	2	22.12%	Immune response-regulating cell surface receptor signaling pathway involved in phagocytosis; Adaptive immune response; Leukocyte mediated immunity	b
B2R4R0	Histone H4	2	21.36%	Cellular Response to stress	b
B7Z992	Gelsolin	2	4.48%	Regulation of leukocyte activation; Regulation of Response to stress	b
P00751	Complement factor B	2	2.36%	Complement activation; Regulation of complement activation	a
P02775	Platelet basic protein	2	14.84%	Antimicrobial Humoral immune response mediated by antimicrobial peptide; Inflammatory response; Immune response;	17
P03950	Angiogenin	2	10.20%	Innate immune response	18

[1-18] Refer to previous literatures

[a] Refer to Uniport database (www.uniport.org)

[b] Refer to gene ontology (GO) analysis

UniquePepCount: the total number of unique peptides associated with the protein group (i.e. these peptides are not shared with another protein group), CoverPercent: sequence coverage of protein

Supplementary Table 3. List of detailed immune system processes of plasma protein identified by LC-MS/MS after exposure to BPNSs.

Uniprot accession number	Protein ID	Unique PepCount	Cover Percent	Biologial process	ref
P02768	Serum albumin	50	80.13%	Toxic substance binding; Rresponse to external stimulus; Response to stress	1, b
P01023	Alpha-2-macroglobulin	22	23.68%	Negative regulation of complement activation, lectin pathway	4
P01024	Complement C3	19	17.86%	Complement activation; Immune response; Neutrophil degranulation; Inflammatory response	2,3
Q53H26	Transferrin variant	15	27.65%	Cellular response to stimulus; Response to stress	b
P00738	Haptoglobin	14	38.92%	regulation of intestinal barrier function	5
D3DNU8	Kininogen 1, isoform CRA_a	12	26.70%	defense response; Regulation of Response to stress	b
P02671	Fibrinogen alpha chain	11	14.32%	facilitate the immune response via both innate and T-cell mediated pathways	6
C0JYY2	Apolipoprotein B (Including Ag(X) antigen)	11	4.38%	regulation of nitric oxide biosynthetic process	b
Q6GMX6	IGH@ protein	10	30.75%	Leukocyte mediated immunity; Immune response-regulating signaling pathway; Complement activation	b
V9HVY1	Epididymis secretory sperm binding protein	10	29.53%	Humoral immune response; response to	b

	Li 78p			cytokine	
Q6N093	Putative uncharacterized protein DKFZp686I04196	8	24.46%	Immune response-regulating cell surface receptor signaling pathway involved in phagocytosis; Leukocyte mediated immunity; Immune response-regulating signaling pathway; Complement activation	b
P02647	Apolipoprotein A-I	8	29.96%	negative regulation of Inflammatory response; negative regulation of response to cytokine stimulus	9
Q6MZX7	Putative uncharacterized protein DKFZp686M24218	7	21.64%	Leukocyte mediated immunity; Adaptive immune response; Complement activation; Immune response-regulating signaling pathway	b
Q8NCL6	Highly similar to Ig alpha-1 chain C region	7	25.56%	positive Regulation of immune system process; Complement activation	b
P04004	Vitronectin	7	12.76%	Regulation of complement activation; immune response	19
Q6PJF2	IGK@ protein	6	42.98%	Humoral immune response; Complement activation; Positive Regulation of immune response	b
Q6P5S8	IGK@ protein	6	42.80%	Humoral immune response; Complement activation; Positive Regulation of immune response	b
A0A0G2JL54	Complement C4-B	6	6.42%	Complement activation; Inflammatory response	a
C9JEU5	Fibrinogen gamma chain	6	18.43%	Platelet activation	a
P02765	Alpha-2-HS-glycoprotein	5	17.71%	Regulation of Inflammatory response	20
A0A075B6N8	Ig gamma-3 chain C region	5	13.53%	B cell receptor signaling pathway; Complement activation; Innate immune response; Positive regulation of B cell activation	21
Q8N355	IGL@ protein	5	29.06%	Positive Regulation of immune system process; Immune response-regulating cell surface receptor signaling pathway involved in phagocytosis; Humoral immune response	b
A0A5E4	Uncharacterized protein	5	28.94%	Humoral immune response; Complement activation; Immune response-regulating signaling pathway	b
P02790	Hemopexin	5	15.80%	Positive regulation of immunoglobulin production; Positive regulation of Humoral immune response mediated by circulating immunoglobulin	a
Q86TT1	Full-length cDNA clone CS0DD006YL02 of	4	12.53%	Immune response	b

B2R8I2	Neuroblastoma of Homo sapiens (human) Highly similar to Homo sapiens histidine-rich glycoprotein (HRG), mRNA	3	8.38%	Regulation of response to external stimulus; Immune response to tumor cell	[b]
S6BGF5	IgG H chain	3	15.42%	Humoral immune response; Complement activation; Immune response-regulating signaling pathway	[b]
A2KBC8	Anti-TeTox scFv	2	14.71%	Leukocyte mediated immunity; Immune response-regulating signaling pathway;	[b]
Q9UL78	My in-reactive immunoglobulin light chain variable region	2	31.19%	Immune response-regulating signaling pathway; Complement activation; Immune response-regulating cell surface receptor signaling pathway	[b]
P01031	Complement C5	2	2.63%	Complement activation; Negative regulation of macrophage chemotaxis; Inflammatory response	[22,23]
P02751	Fibronectin	2	1.05%	Leukocyte migration	[a]
A0A0A0MS51	Gelsolin	2	5.35%	Regulation of leukocyte activation; Lymphocyte activation; Regulation of Response to stress	[b]
A0A109PW65	GCT-A3 light chain variable region	2	31.78%	Leukocyte mediated immunity; Immune response-regulating signaling pathway; Complement activation	[b]
A0A125QYY5	GCT-A9 light chain variable region	2	31.78%	Leukocyte mediated immunity; Immune response-regulating signaling pathway; Complement activation	[b]
B2R4R0	Histone H4	2	21.36%	Cellular Response to stress	[b]
E7ETN3	Uncharacterized protein	2	2.87%	Innate immune response; Complement activation; Positive Regulation of immune response	[b]
P02748	Complement component C9	2	6.98%	Regulation of complement activation	[a]
P10909	Clusterin	2	4.23%	Complement activation; Innate immune response	[24]
P19652	Alpha-1-acid glycoprotein 2	2	9.95%	Regulation of immune system process	[a]

[1-6, 9, 19-24] Refer to previous literatures

[a] Refer to Uniport database (www.uniport.org)

[b] Refer to gene ontology (GO) analysis

UniquePepCount: the total number of unique peptides associated with the protein group (i.e. these peptides are not shared with another protein group), CoverPercent: sequence coverage of protein

Supplementary References

1. Ghuman, J. *et al.* Structural basis of the drug-binding specificity of human serum albumin. *J. Mol. Biol.* **353**, 38-52 (2005).
2. Gullstrand, B., Mårtensson, U., Sturfelt, G., Bengtsson, A. A. & Truedsson, L. Complement classical pathway components are all important in clearance of apoptotic and secondary necrotic cells. *Clinical & Experimental Immunology* **156**, 303-311 (2009).
3. Alper, C. A., Abramson, N., Jr, J. R., Jandl, J. H. & Rosen, F. S. Studies in vivo and in vitro on an abnormality in the metabolism of C3 in a

patient with increased susceptibility to infection. *Journal of Clinical Investigation* **49**, 1975 (1970).
4. Ambrus, G. *et al.* Natural substrates and inhibitors of mannan-binding lectin-associated serine protease-1 and -2: a study on recombinant catalytic fragments. *Journal of Immunology* **170**, 1374-1382 (2003).
5. Fasano, A. Zonulin and its regulation of intestinal barrier function: the biological door to inflammation, autoimmunity, and cancer. *Physiological Reviews* **91**, 151-175 (2011).
6. Luo, D. *et al.* Fibrin facilitates both innate and T cell-mediated defense against Yersinia pestis. *Journal of Immunology* **190**, 4149-4161 (2013).
7. Petrušić, V. *et al.* Antigenic specificity and expression of a natural idiotope on human pentameric and hexameric IgM polymers. *Immunologic Research* **51**, 97-107 (2011).
8. Fleury, C. *et al.* Identification of a Haemophilus influenzae factor H-Binding lipoprotein involved in serum resistance. *Journal of Immunology* **192**, 5913 (2014).
9. Di, B. B. *et al.* The apolipoprotein A-I mimetic peptide ETC-642 exhibits anti-inflammatory properties that are comparable to high density lipoproteins. *Atherosclerosis* **217**, 395-400 (2011).
10. Skerka, C., Horstmann, R. D. & Zipfel, P. F. Molecular cloning of a human serum protein structurally related to complement factor H. *J. Biol. Chem.* **266**, 12015-12020 (1991).
11. Gebe, J. A. *et al.* Molecular cloning, mapping to human chromosome 1 q21-q23, and cell binding characteristics of Spalpha, a new member of the scavenger receptor cysteine-rich (SRCR) family of proteins. *J. Biol. Chem.* **272**, 6151 (1997).
12. Gorgani, N. N., Parish, C. R. & Altin, J. G. Differential binding of histidine-rich glycoprotein (HRG) to human IgG subclasses and IgG molecules containing kappa and lambda light chains. *J. Biol. Chem.* **274**, 29633-29640 (1999).
13. Max, E. E. & Korsmeyer, S. J. Human J chain gene. Structure and expression in B lymphoid cells. *J. Exp. Med.* **161**, 832-849 (1985).
14. Petrušić, V. *et al.* Antigenic specificity and expression of a natural idiotope on human pentameric and hexameric IgM polymers. *Immunologic Research* **51**, 97 (2011).
15. López-Lira, F., Rosales-León, L., Martínez, V. M. & Ruiz Ordaz, B. H. The role of beta2-glycoprotein I (beta2GPI) in the activation of plasminogen. *BBA* **1764**, 815 (2006).
16. Petry, F. Molecular Basis of Hereditary C1q Deficiency. *Immunobiology* **199**, 286-294, doi:https://doi.org/10.1016/S0171-2985(98)80033-8 (1998).
17. Krijgsveld, J. *et al.* Thrombocidins, microbicidal proteins from human blood platelets, are C-terminal deletion products of CXC chemokines. *J. Biol. Chem.* **275**, 20374 (2000).
18. Hooper, L. V., Stappenbeck, T. S., Hong, C. V. & Gordon, J. I. Angiogenins: a new class of microbicidal proteins involved in innate immunity. *Nat. Immunol.* **4**, 269 (2003).
19. Preissner, K. T., Heimburger, N., Anders, E. & Müller-Berghaus, G. Physicochemical, immunochemical and functional comparison of human S-protein and vitronectin evidence for the identity of both plasma proteins. *Biochemical & Biophysical Research Communications* **134**, 951-956 (1986).
20. Ketteler, M. *et al.* Association of low fetuin-A (AHSG) concentrations in serum with cardiovascular mortality in patients on dialysis: a cross-sectional study. *Lancet* **361**, 827-833 (2003).
21. Mcheyzerwilliams, M., Okitsu, S., Wang, N. & Mcheyzerwilliams, L. Molecular programming of B cell memory. *Nat. Rev. Immunol.* **12**, 24 (2011).
22. Nilsson, G. *et al.* C3a and C5a are chemotaxins for human mast cells and act through distinct receptors via a pertussis toxin-sensitive signal transduction pathway. *Journal of Immunology* **157**, 1693-1698 (1996).
23. J, A. *et al.* An animal model of age-related macular degeneration in senescent Ccl-2- or Ccr-2-deficient mice. *Nat. Med.* **9**, 1390-1397 (2003).
24. Jenne, D. E. & Tschopp, J. Clusterin: the intriguing guises of a widely expressed glycoprotein. *Trends Biochem. Sci* **17**, 154 (1992).
