# Supplementary material for: Revealing the immune perturbation of black phosphorus nanomaterials to macrophages by understanding the protein corona
Source: Nat Commun. 2018 Jun 26;9:2480. doi: 10.1038/s41467-018-04873-7 (PMC6018659; doi:10.1038/s41467-018-04873-7)
Supplement: Supplementary file 3 — Description of Additional Supplementary Files [file 41467_2018_4873_MOESM3_ESM.pdf]

Description of Additional Supplementary Files

File Name: Supplementary Data 1
Description: List of plasma protein identified by LC-MS/MS (from 3 parallel experiments) after exposure to BPNSs.

File Name: Supplementary Data 2
Description: List of plasma protein identified by LC-MS/MS (from 3 parallel experiments) after exposure to BPQDs.

File Name: Supplementary Data 3
Description: List of plasma protein identified by LC-MS/MS (from 3 parallel experiments).
